# Characterization, Source Analysis, and Ecological Risk Assessment of Heavy Metal Pollution in Surface Soils from the Central–Western Ali Region on the Tibetan Plateau

**DOI:** 10.3390/toxics13110972

**Published:** 2025-11-12

**Authors:** Yanping Huang, Tieguang He, Jun Luo, Xueyang Ma, Tuo Zhang

**Affiliations:** 1 College of Environmental Science and Engineering, China West Normal University, Nanchong 637009, China; 13158581039@139.com; 2 Agricultural Resources and Environmental Research Institute, Guangxi Academy of Agricultural Sciences/Guangxi Key Laboratory of Arable Land Conservation, Nanning 530007, China; tghe118@163.com; 3 School of Geographical Sciences, China West Normal University, Nanchong 637009, China; luojunxmx@126.com; 4 School of Environmental and Life Science, Nanning Normal University, Nanning 530001, China

**Keywords:** surface soil, heavy metals, ecological risk assessment, source apportionment, Tibetan Plateau

## Abstract

Most risk assessment and source apportionment studies of the heavy metals in the surface soils in China have focused primarily on East China, whereas studies focused on Northwest China, particularly regarding heavy metals in surface soils in the central and western areas, remain limited. In this study, surface soils in the central–western Ali region were investigated, and the concentrations of nine heavy metals were determined. Moreover, the distribution patterns and ecological risks of these heavy metals were elucidated via a combination of the geoaccumulation index, pollution load index (PLI), comprehensive potential ecological risk index (RI), and integrated X-ray diffraction (XRD)–multivariate statistical techniques. Additionally, the pollution characteristics and sources were analyzed. The results indicated the following: (1) The spatial distribution of heavy metal pollution is closely linked to the geological background, and high–pollution zones (e.g., Cr, Ni, Co, Cu, As, and Cd) conform well with the distributions of ultramafic rocks and iron/chromite ore beds. The geoaccumulation index revealed that Cd caused slight and moderate contamination at 29.1% and 5.5% of the sites, respectively, whereas As affected 14.6% of the sites. The pollution load index indicated moderate pollution in 20% of the sites, and the potential ecological risk index indicated that 41.8% of the sites posed moderate risks, which was largely driven by Cd (mean $E_{r}^{i}$ = 43.1). The comprehensive ecological risk index (RI = 115) confirmed a moderate risk level overall. Principal component analysis revealed three primary sources: natural weathering (Cr–Ni–Co–Cu, 39.1%); a mixed source influenced by nonagricultural anthropogenic activities such as transport and regional deposition, combined with natural processes such as arid climate and alkaline soil conditions that influence Cd mobility (Cd–Mo–Pb, 20.8%); and industrial/mining activities (As–Sb, 14.2%). Mineralogical analyses further indicated that heavy metals are present via lattice substitution, adsorption, and precipitation. This study systematically clarifies the composite pollution pattern and sources of heavy metals in the alpine Ali region, supporting targeted contamination control.

## 1. Introduction

Heavy metals refer to a group of metals whose specific gravities are greater than 4.5 g·cm^−3^ and primarily include elements such as Hg, Cd, Pb, Cu, Cr, As, Ni, Fe, Mo, and Zn. Although As is not strictly classified as a heavy metal, owing to its similar behavior, sources, and hazards, it is commonly considered alongside heavy metals [1]. Among these heavy metals, Cd, Cr, Cu, Pb, Ni, and Zn have been designated major regulated pollutants by the U.S. Environmental Protection Agency (USEPA) [2], mainly because of their persistence in soils that is characterized by long residence times, high concealment, high toxicity, low self–purification capacity, and notable resistance to biodegradation [3,4]. Serving as the central hub for material cycling and energy flow within ecosystems, soil heavy metals can be absorbed by plants and enter the food chain, ultimately posing a threat to human health [5]. Therefore, investigating the distribution and source mechanisms of heavy metals in surface soils is crucial for ensuring ecological safety and public health protection.

The impacts of heavy metal pollution extend far beyond emission source areas and can lead to regional or even global–scale issues through atmospheric transport [6,7,8]. Since the Industrial Revolution, global Hg accumulations in organic soil layers have increased by approximately 20% because of anthropogenic emissions [9], whereas Pb accumulations in remote northern European lake sediments have increased by 1–5 g·m^−2^ [10]. High–altitude regions are of particular concern; influenced by the cold trap effect, middle– to high–altitude mountains serve as significant sinks for heavy metals in the atmosphere [11,12], and their fragile ecosystems are especially sensitive to pollution [13,14]. Research has focused primarily on economically developed regions in East China (e.g., Shandong, Jiangsu, Anhui, Shanghai, Zhejiang, and Fujian), whereas studies in Northwest China have focused mainly on Shaanxi, Gansu, and Xinjiang [15], with a notable lack of attention given to key ecological zones, such as the Ali region.

The central–western Ali region is a vital biogenetic reservoir within China’s northwestern desert area and has significant strategic ecological importance. This region supports the unique flora and fauna of the Tibetan Plateau and serves as a core component of the ecological security barrier of the plateau. However, its stability faces threats from both geological and climatic factors. Located in the India–Eurasia Plate collision zone, the weathering of ultramafic rocks and sulfide ore beds in this area can result in the release of substantial amounts of Ni, Cr, and other heavy metals [16]. Moreover, the regional arid climate with low annual precipitation (<100 mm) limits leaching processes [17], while the reduced microbial activity under cold conditions slows organic matter degradation, in turn impeding natural heavy metal purification [18]. Additionally, the Tibetan Plateau is experiencing warming at a rate significantly higher than the global average [19], which may trigger the release of historically deposited heavy metals because of permafrost degradation. Glacial retreat can also facilitate the transport of pollutants downstream via meltwater [20]. This coupled geological release–climate–driven mechanism complicates the pollution dynamics in the Ali region compared with those in low–elevation industrial areas, necessitating systematic research to elucidate migration and transformation patterns.

In this study, a “geological background–mineral occurrence–environmental factor” coupling model is established, and multiple methods, including the geoaccumulation index, pollution load index, potential ecological risk index, and XRD–multivariate statistical analysis, are integrated to systematically investigate the spatial distribution, sources, and dominant control mechanisms of nine heavy metals in surface soils from the central–western Ali region. This research focuses on elucidating the regulatory role of mineral phases (e.g., spinel, sulfides, and silicates) in controlling heavy metal speciation, identifying the contributions of natural weathering and anthropogenic inputs, assessing ecological risks, and proposing targeted heavy metal pollution control strategies for the alpine ecological barrier zone. These findings aim to fill critical knowledge gaps regarding heavy metal behaviors and source–sink mechanisms in this understudied area.

## 2. Materials and Methods

### 2.1. Overview of the Study Area

**Figure 1:**
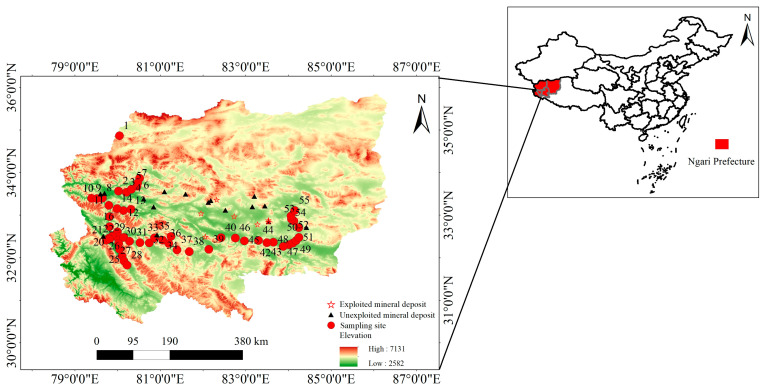
Spatial distribution of the surface soil sampling sites in the central and western Ali region.

The Ali region is located northwest of the autonomous region, at the core of the northern part of the Tibetan Plateau, featuring high terrain and an average altitude of approximately 4663 m. This region is located at the junction of the Himalayas and Kunlun Mountains and is the source of multiple rivers inside and outside the territory. Therefore, the central–western Ali region occupies an extremely important ecological strategic position, with a plateau subregion climate that is characterized by cold and dry conditions, an annual precipitation lower than 50–100 mm, and an annual average relative humidity ranging from 30 to 40%, which can be classified as consisting of high–cold meadow and grassland areas; this ecosystem is extremely fragile [21]. The Ali region contains abundant mineral resources. Notably, there are currently 29 boron deposits of national industrial grade, 130 gold deposits (beds and points), 20 copper deposits, and 11 iron deposits. The mineral deposits in the area are predominantly unexploited and include magnetite and copper–gold in Gerze County; magnetite and chromite in Ritu County; iron ore in Gar County and magnetite, iron ore, lead–zinc, and copper–gold in Geji County. Exploited deposits in Geji and Gaize County include lead–zinc deposits, plumbian iron deposits, and copper and gold deposits. The distributions of these deposits are shown in Figure 1.

### 2.2. Sampling and Analysis

In September 2020, during periods of no dust activity and minimal wind speeds, surface soil samples were collected from 55 sampling points arranged along an east–west transect at the southern margin of the Kunlun–Altun Mountains. Each sample was obtained from the top 0–10 cm of soil, with approximately 500 g collected per site. The concentration data are reported in mg·kg^−1^ (dry weight). The locations of the sampling points are shown in Figure 1, with efforts made to avoid areas with significant human disturbances.

After collection, the samples were carefully preserved and transported, after which they were dried naturally and sieved through a 100–mesh sieve. The air–dried and sieved surface soil samples were pretreated via digestion using a mixed acid solution (HNO_3_–HClO_4_–HF) on an electric hot plate, following national standard protocols. Notably, this digestion scheme, by avoiding the use of hydrochloric acid (HCl), effectively minimizes the introduction of exogenous chlorine ions, thereby significantly reducing the potential for polyatomic interference on arsenic at the source. The digested samples were analyzed via inductively coupled plasma–mass spectrometry (ICP–MS). ICP–MS analyses were performed on an instrument equipped with a collision/reaction cell (CRC). To specifically address the critical spectral interference of ^40^Ar^35^Cl^+^ on ^75^As^+^, the instrument was operated in reaction mode with oxygen (O_2_) as the cell gas. Under this mode, ^75^As^+^ reacts to form ^75^As^16^O^+^ (*m*/*z* 91), which is then measured and is free from the original chloride–based interference.

To ensure the accuracy and precision of the data, a comprehensive quality assurance/quality control (QA/QC) protocol was strictly implemented. This included analyses of certified reference materials (CRMs), procedural blanks, and sample duplicates for every batch of samples. The recovery rates for the target heavy metals in the CRMs were consistently within the acceptable range of 85–115%. The relative percentage difference (RPD) between duplicates was typically less than 10%. Furthermore, a mixed internal standard containing indium (In), rhodium (Rh), and rhenium (Re) at a concentration of 10 μg·L^−1^ was introduced online to correct for instrumental drift and matrix suppression effects during the entire ICP–MS analytical process. A known concentration of heavy metal solution (20 μg·L^−1^) was also used as an external standard. Throughout the detection process, the recoveries of the internal and external standards were greater than 96.5% and 98.6%, respectively. In summary, through a combination of a chlorine–minimized digestion procedure, advanced CRC instrumental technology, and rigorous QA/QC measures, the analytical data for heavy metals were ensured to be highly reliable and accurate.

The pH and total nitrogen (TN) contents of the air–dried soil samples were determined using the glass electrode method and the Kjeldahl nitrogen method, respectively, following standard procedures. Total potassium (TK), total organic carbon (TOC), and total phosphorus (TP) contents were measured by flame photometry, a TOC analyzer, and the antimony tartrate spectrophotometric method, respectively. In this study, TK serves as an important indicator for assessing soil fertility, as potassium is an essential nutrient for plant growth, and its content reflects the potential potassium supply capacity of the soil in the study area. Additionally, the soil carbon–to–nitrogen ratio (C/N) was calculated as the ratio of total carbon to total nitrogen content, indicating the relative proportion of carbon and nitrogen.

The mineralogical compositions were determined via X-ray powder diffraction (XRD) with a Rigaku Ultima IV diffractometer. Phase identification was conducted by matching diffraction patterns to the Crystallography Open Database (COD) with Rietveld refinement software on the basis of the peak positions and intensities, and the relative content of each mineral phase was estimated through peak area calculations.

### 2.3. Evaluation of Heavy Metal Pollution

The geoaccumulation index method is a mathematical model that is based on the modification of background matrices and is widely employed for quantitative evaluations of soil heavy metal pollution [22]. The geoaccumulation index can be expressed as follows:

$$I_{geo}={log}_{2}(\frac{C_{n}}{1.5B_{n}})$$

where $C_{n}$ denotes the heavy metal content at the site and $B_{n}$ denotes the geochemical background value of the heavy metal; the index evaluation classification is detailed in Table S1 [22].

The pollution load index (PLI) method, proposed by Tomlinson [23], reflects both the pollution levels of individual contaminants and their contributions to the overall pollution status. This index is widely used for assessing heavy metal pollution in site and farmland soils. The PLI can be calculated as follows:

$$CF=C_{sample}^{i}/C_{background}^{i}$$

$$PLI={\left(CF_{1}\times CF_{2}\times CF_{3}\times CF_{n}\right)}^{1/n}$$

where $C_{sample}^{i}$ denotes the measured of heavy metal content, mg·kg^−1^; $C_{background}^{i}$ denotes the background heavy metal value, mg·kg^−1^; and CF denotes the contamination factor. PLI > 1 indicates the occurrence of heavy metal pollution [23]. Details are provided in Table S1 [24].

### 2.4. Heavy Metal Risk Assessment

The potential ecological risk index ($E_{r}^{i}$) can be used to assess the degree and potential risk of heavy metal pollution. Notably, the heavy metal pollution status can be evaluated more accurately by determining the toxicity coefficients of individual heavy metals and applying this index [25].

$$C_{r}^{i}=C_{i}/C_{n}^{i}$$

$$E_{r}^{i}=T_{r}^{i}\times C_{r}^{i}$$

$$RI=\sum_{i=1}^{n}E_{r}^{i}$$

where $C_{i}$ denotes the content of heavy metal *i* in the soil, mg·kg^−1^; $C_{n}^{i}$ denotes the background value of heavy metal i in the soil, mg·kg^−1^; $C_{r}^{i}$ denotes the enrichment factor of heavy metal *i* in the soil; and $T_{r}^{i}$ denotes the toxicity coefficient of heavy metal *i* in the soil. The toxicity coefficients are as follows: Co = Ni = Cu = Pb = 5, Cd = 30, Cr = 2, As = 10, Sb = 40, and Mo = 15 [26,27]. Moreover, $E_{r}^{i}$ denotes the potential ecological risk index of heavy metal *i* in the soil, and *RI* denotes the comprehensive potential ecological risk index for multiple heavy metals in the soil. The calculation results were classified according to Table S2 [28].

The single–factor pollution index refers to the ratio of the measured content of a given heavy metal in the soil to its evaluation criterion, directly reflecting the extent of pollution, with an index value that is proportional to the degree of pollution [29]. The single–factor pollution index can be calculated as follows:

$$P_{i}=C_{s}^{i}/C_{n}^{i}$$

where $P_{i}$ is the single–factor pollution index of a given heavy metal in the soil; $C_{s}^{i}$ is the measured content, mg·kg^−1^; and $C_{n}^{i}$ is the associated evaluation criterion, mg·kg^−1^. Here, the background value of the soil elements was selected as the limit value [30].

In recent years, the Nemerow integrated pollution index method has been widely applied in research on heavy metal soil pollution, primarily because it can reasonably reflect the comprehensive degree of pollution in the study area [31,32]. The maximum (max) and average (ave) single–factor pollution index values of all the heavy metals were used to determine the composite pollution index, which can be expressed as follows:

$$P=\sqrt{(P_{i,ave}^{2}+P_{i,max}^{2})/2}$$

where P is the Nemerow integrated pollution index, $P_{i,ave}^{2}$ is the average value of the single–factor pollution index, and $P_{i,max}^{2}$ is the maximum value of the single–factor pollution index. Table S2 provides the evaluation grades of the single–factor pollution index [33] and the Nemerow integrated pollution index [34].

## 3. Results

### 3.1. Semiquantitative Surface Soil XRD

Semiquantitative XRD analysis of the surface soil samples indicated that the mineral compositions in the central–western Ali region are dominated by quartz and feldspar, suggesting that the soil primarily originates from the weathering of silicate minerals. On the basis of the semiquantitative XRD data, we performed hierarchical cluster analysis using Euclidean distance and Ward’s linkage methods to objectively classify the mineral assemblages in our samples. The optimal number of clusters was determined to be five by drawing a horizontal line across the dendrogram at a rescaled distance of 15 (Figure S1). To intuitively present the results of this classification, Figure S2 visually summarizes the mineral composition and relative abundance for each of the five identified clusters. Each cluster was named according to its dominant mineral constituents (>10% content) and the presence of characteristic minerals, such as sulfides or carbonates: Classification 1 (silicate–sulfide–uranium mineral assemblage): primarily composed of quartz, feldspar, and other silicates, accompanied by sulfides and uranium minerals; Classification 2 (silicate–carbonate assemblage): characterized by a mixture of silicates (e.g., quartz and feldspar) and carbonates such as calcite and dolomite; Classification 3 (silicate–cadmium–arsenic mineral assemblage): dominated by silicates but also containing cadmium minerals (e.g., otavite) and arsenic minerals (e.g., arsenpolybasite); Classification 4 (silicate–pyroxene assemblage): mainly consisting of silicates (e.g., quartz and feldspar) and pyroxenes; Classification 5 (silicate–oxysalt–graphite assemblage): defined by the coexistence of silicates, oxysalt minerals (such as sulfates), and graphite. Previous studies have demonstrated that soil mineral compositions play crucial roles in influencing the migration and accumulation of heavy metals [35]. Therefore, understanding the relationship between mineralogy and heavy metal pollution is essential.

### 3.2. Distribution of Heavy Metals in Surface Soil

The pH of the surface soils in the central–western Ali region ranged from 7.8 to 10.1, with an average of 8.8. TN contents varied between 0.1 and 1.1 g/kg, with an average of 0.4 g/kg; TOC contents ranged from 1.0 to 68.4 g/kg, with an average of 14.3 g/kg; TP contents ranged from 0.2 to 0.8 g/kg, with an average of 0.4 g/kg; the C/N ratios ranged from 22.1 to 147, with an average of 67.2; and TK contents ranged from 2.5 to 10.1 g/kg, with an average of 5.4 g/kg. These results indicate an uneven distribution of soil nutrients across the central–western Ali region.

The descriptive statistics for the heavy metals in surface soils from the central–western Ali region are summarized in Table 1. The average concentrations of the nine elements decrease in the order of Cr > Ni > As > Pb > Cu > Co > Sb > Mo > Cd. Compared with the background values [30], the average Cr, Ni, As, and Cd concentrations are slightly elevated, whereas those of the other elements are lower. Compared with the values for the upper continental crust (UCC) [36], the surface soils showed a distinct sequence of enrichment: Cr, Ni, and As were relatively enriched; Cd, Sb, and Pb were present at similar levels; and Co, Cu, and Mo were depleted. Compared with the risk–based screening thresholds [37], the average Cr, Co, Ni, Cu, As, Mo, Cd, Sb, and Pb concentrations were lower. However, the maximum values of Cr and As indicated local exceedances, with Cr exceeding the threshold at 3.6% of the sites and As exceeding the threshold at 23.6% of the sites. For example, site XZ–55 near Rena Cuo Lake in Gaize County exhibited As concentrations up to seven times the regulatory limit.

In the central–western Ali region, the coefficient of variation (CV) of the heavy metals in the surface soils followed the order of As > Cr > Ni > Sb > Cd > Cu > Mo > Co > Pb. Among these elements, As exhibited extremely high variability (>100%), whereas Cr, Ni, Cd, and Sb showed high variability (50–100%). This high spatial heterogeneity suggests that these five elements may be influenced by point source pollution. The exceptionally high variability of As and Sb could be further amplified by their high mobility under the alkaline conditions that are typical of the study area, which facilitate their translocation and redistribution [38]. The remaining elements demonstrated moderate variability (20–50%). Additionally, the elements with high CV values demonstrated greater skewness and kurtosis values, thus supporting the hypothesis that human activities are the primary drivers of spatial heterogeneity. Therefore, the concentrations of Cr, Ni, Cd, and As in the surface soils from the central–western Ali region are significantly affected by anthropogenic factors, with certain sites experiencing severe As contamination.

### 3.3. Assessment of Heavy Metal Pollution

#### 3.3.1. Geoaccumulation Index ($I_{geo}$)

**Figure 2:**
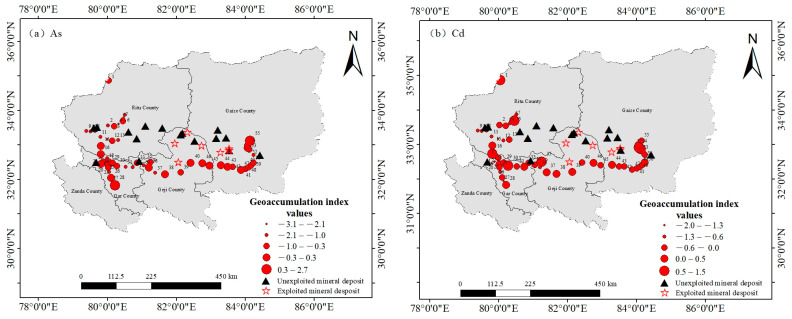
Spatial distributions of the geoaccumulation indices of heavy metals in surface soils in the central and western Ali region ($I_{geo}$). (**a**) As, (**b**) Cd.

According to the assessment results for the geoaccumulation index, the surface soils in the central–western Ali region exhibited varying degrees of heavy metal pollution, ranging from uncontaminated to moderately and heavily contaminated levels ($I_{geo}$ values ranging from −3.2 to 2.7) (Figure 2). To comprehensively reflect the environmental background status of all the investigated heavy metals, the mean $I_{geo}$ values are reported here even when they are negative (indicating no contamination). The pollution intensities were ranked in descending order as follows: Cd (−0.2) > As (−0.7) = Ni (−0.7) > Cr (−0.8) > Sb (−0.1) > Pb (−1.0) > Co (−1.1) > Mo (−1.3) > Cu (−1.4). These negative values provide important baseline information, indicating that these elements are at or below the natural background levels in the local soil, which further confirms the overall low contamination levels in the study area. Among the heavy metals studied, although the mean $I_{geo}$ value for Cd indicated no overall contamination, localized enrichment was evident, with 29.1% and 5.5% of the sampling sites categorized as slightly or moderately contaminated, respectively. The $I_{geo}$ values for arsenic (As) varied widely (−3.1 to 2.7), and the mean value (−0.7) also indicated an overall absence of contamination. However, localized enrichment was observed at a small number of sites, with 10.9%, 1.8%, and 1.8% of the sites classified as slightly, moderately, or moderately to heavily contaminated, respectively. These results demonstrate that although the heavy metal pollution in the study area is generally not severe, localized contamination does occur. Therefore, continuous attention should be given to the dynamics of heavy metal pollution in the region to prevent further spread.

The spatial distributions of the geoaccumulation index ($I_{geo}$) are presented in Figure 2 for As and Cd and in Figure S3 for the remaining heavy metals, revealing distinct localized hotspots with composite pollution. Pollution levels were more severe in the eastern and western parts of the central–western Ali region, where the As and Cd levels were slightly higher. With respect to the other seven heavy metals, more than 80% of the sites could be classified as uncontaminated. Considering the distribution of ore deposits, this pattern may be linked to mineral occurrences in these areas. The data from sites XZ–8, XZ–10, XZ–21, XZ–33, XZ–39, and XZ–51 were obtained near magnetite, chromite, iron ore, copper–gold, and copper–molybdenum deposits, respectively (Figure 1). Although the geoaccumulation index values are elevated near these mineral deposits, most remain unexploited, suggesting that the observed pollution may result from natural mineral migration and enrichment processes. Consequently, the geoaccumulation index values generally remain low, with only a few sites demonstrating high pollution levels.

#### 3.3.2. Pollution Load Index (PLI)

**Figure 3:**
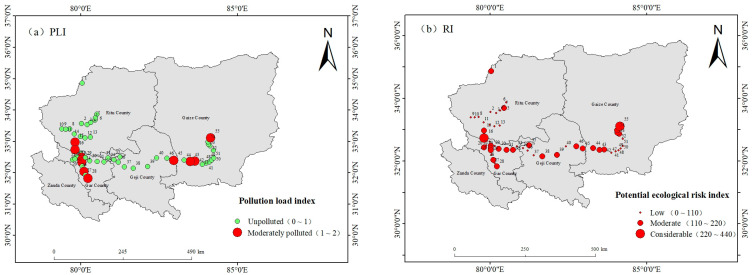
Spatial distributions of the pollution load index and potential ecological risk index. (**a**) *PLI* and (**b**) *RI*.

The spatial distribution map of the pollution load index (PLI) for the central–western Ali region was generated using ArcGIS 10.8 software (Figure 3a). The results indicated that 20% of the surface soil sampling sites in the region could be categorized as moderately polluted and were primarily concentrated in the eastern and western areas. This pollution pattern closely aligns with the Cd contamination zones that were identified on the basis of the geoaccumulation index. Although 80% of the sampling sites were in the unpolluted category (Figure 3b), the potential ecological risk index (RI) revealed that 41.8% of the sites exhibited moderate risk. This suggests that despite the extremely sparse population of the central–western Ali region, the issue of heavy metal pollution should not be overlooked.

### 3.4. Heavy Metal Risk Assessment

#### 3.4.1. Potential Ecological Risk Index ($E_{r}^{i}$)

**Figure 4:**
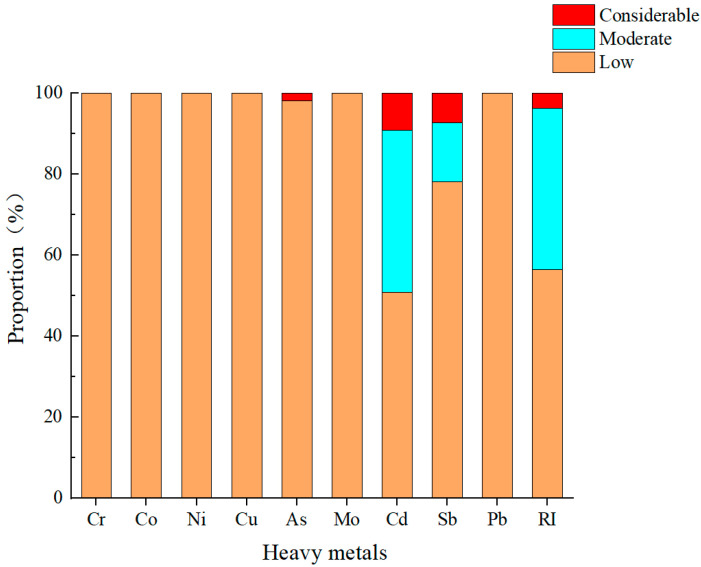
Proportions of sampling sites at different pollution levels for heavy metals in surface soil in the central and western Ali region (%).

The values for the potential ecological risk index ($E_{r}^{i}$) of the heavy metals in the surface soils at the different sampling sites in the central–western Ali region are shown in Figure 4. A comparison of the ecological risk index of the various heavy metals revealed that, except for As, Cd, and Sb, the other six heavy metals posed only low ecological risks across all the sampling points. With respect to Cd and Sb, 40% and 14.6% of the sampling sites, respectively, were within the moderate ecological risk category. Moreover, 1.8%, 9.1%, and 7.3% of the sites exhibited considerable ecological risks from As, Cd, and Sb, respectively. Through the analysis of the overall risk distribution based on the comprehensive potential ecological risk index (RI), 40% of the sampling points in the central–western Ali region could be classified as exhibiting moderate risk, and 3.6% fell into the considerable risk category. The values for the mean ecological risk ($E_{r}^{i}$) of the nine heavy metals in surface soils are as follows: Cr (2.1), Co (3.7), Ni (5.8), Cu (3.2), As (11.9), Mo (9.7), Cd (43.1), Sb (34.6), and Pb (3.6). Among these, only Cd reached the moderate potential ecological risk level, identifying it as the main potential ecological risk factor in the surface soils from the study area, which is consistent with the results of the heavy metal pollution assessment. The values for the comprehensive potential ecological risk index (RI) of the heavy metals in the central–western Ali region ranged from 40.5 to 293, with a mean value of 115, corresponding to a moderate ecological risk level. This finding indicates that despite the extremely sparse population of the region, heavy metal pollution persists, underscoring the need for continued monitoring and research in the central–western Ali region.

#### 3.4.2. Single–Factor Pollution Index and Nemerow Integrated Pollution Index

**Figure 5:**
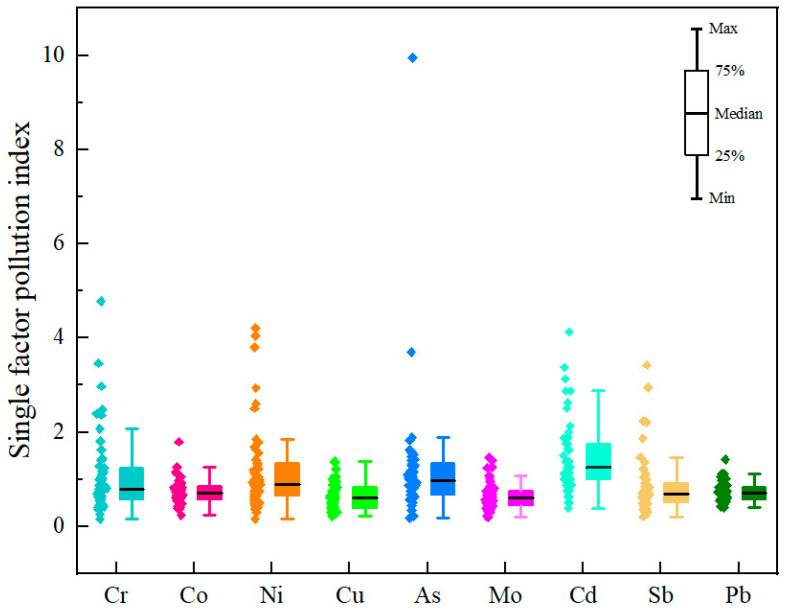
Box plots of the single–factor pollution index for heavy metals in surface soil in the central and western regions of Ali.

The values for the single–factor pollution index of the heavy metals in the surface soils from the central–western Ali region are listed in Figure 5. The results revealed that all nine heavy metals caused varying degrees of pollution. Among them, Cd was the most widely occurring element, with 52.7%, 9.1%, and 5.5% of the sampling points classified as slightly, moderately, or strongly polluted, respectively. The pollution status for As was less severe. However, certain sites exhibited severe contamination, with 43.6%, 1.8%, and 1.8% of the sites classified at the slight, strong, or extreme pollution levels (notably site XZ–55), respectively. The values for the single–factor pollution index of Cr and Ni indicated primarily slight pollution, accounting for 21.8% and 27.3% of the sites, respectively, but moderate and strong pollution levels were also observed at 9.09% and 3.6% (Cr) and 5.5% and 5.5% (Ni) of the sampling points, respectively. With respect to the remaining five heavy metals, more than 78.2% of the sites fell within the unpolluted category. Spatially, slightly polluted areas were moderately concentrated in Gaize County, whereas moderately and strongly polluted areas were primarily located around magnetite, iron ore, and chromite deposits in Ritu and Gar Counties.

The mean values for the single–factor pollution index and the integrated Nemerow pollution index for heavy metals in surface soils from the Ali region are provided in Table S3. According to the Nemerow integrated pollution index, Cr, Ni, As, and Cd could be classified into the heavily polluted category, primarily because their maximum values reached moderately polluted levels. On the basis of the average values, the central–western Ali region generally exhibited a moderately polluted state. By combining the single–factor pollution index with the Nemerow integrated pollution index, it was determined that the Cr, Ni, As, and Cd pollution in the surface soils of Ritu and Gar Counties, as well as the area surrounding Renacuo Lake in Gaize County, warrants special attention. Strengthened control and management measures are necessary to prevent further deterioration of the pollution in these areas.

### 3.5. Correlation Analysis

#### 3.5.1. Correlations of Heavy Metals in the Surface Soils from the Central–Western Ali Region

**Figure 6:**
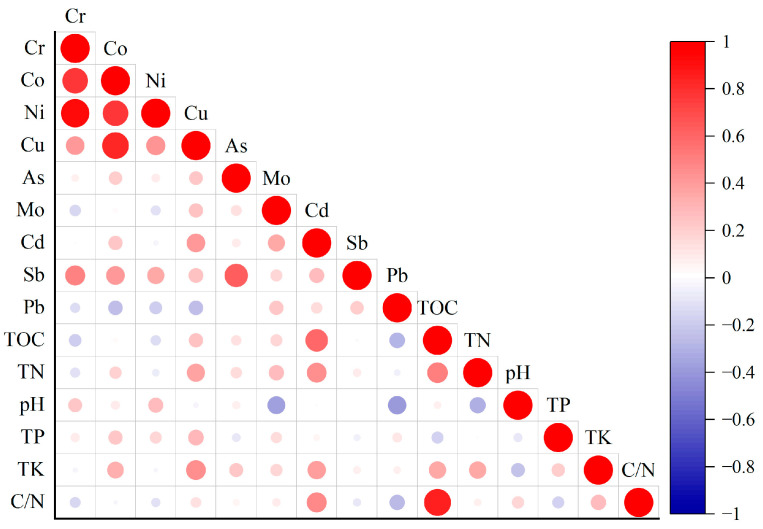
Pearson correlation heatmap of the heavy metals in the surface soils of the central and western regions of Ali.

Correlation analysis revealed key associations between soil properties and heavy metals (Figure 6). Notably, TK, TN, TOC were significantly positively correlated with Cd and Cu, suggesting a potential common source or similar behavior in the soil environment for these metals. In contrast, soil pH was significantly negatively correlated with Mo and Pb, indicating that their availability or retention may decrease under less acidic conditions. Other significant, albeit weaker, correlations are detailed in Figure 6.

With respect to the correlations among the heavy metals themselves, at the *p* < 0.01 level, Cr was highly significantly positively correlated with Co, Ni, Cu, and Sb; Co was highly significantly positively correlated with Ni, Cu, and Sb; Ni was highly significantly positively correlated with Cu and Sb; Cu was highly significantly positively correlated with Cd; and As was highly significantly positively correlated with Sb. At the *p* < 0.05 level, Mo was significantly positively correlated with Cd.

#### 3.5.2. Principal Components of Heavy Metal Elements in the Surface Soils from the Central–Western Ali Region

To identify the sources of heavy metals in the surface soils from the central–western Ali region, principal component analysis (PCA) was conducted on the basis of the correlation results, as detailed in Table S4. The first three principal components with eigenvalues greater than 1 were extracted, which accounted for a cumulative contribution rate of 74.0%. Therefore, the subsequent analysis focuses primarily on these three components, which effectively represent the overall data structure.

As indicated in Table S4 and Figure S4, the first principal component explained 39.1% of the overall variance, with high loadings on Cr, Co, Ni, and Cu. On the basis of the correlation analysis results, these elements could be considered to share similar source pathways. The second principal component accounted for 20.8% of the total variance and was characterized by relatively high loadings of Mo, Cd, and Pb. The third principal component accounted for 13.9% of the total variance and was dominated by As and Sb.

#### 3.5.3. Correlations Between Heavy Metal Element Contents and Mineral Compositions in the Surface Soils from the Central–Western Ali Region

**Figure 7:**
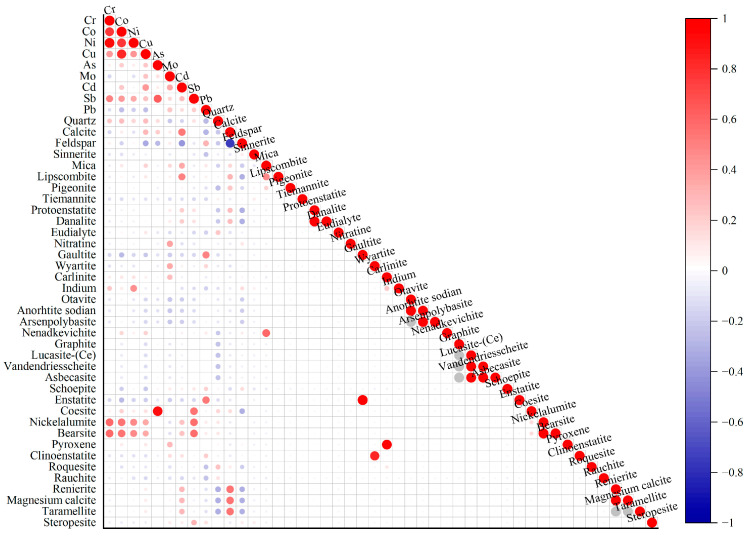
Pearson correlation heatmap of mineral and heavy metal contents.

Semiquantitative XRD analyses were conducted to determine the mineral compositions at the sampling points (Figure 7). At a significance level of *p* < 0.01, Cr, Co, Ni, Cu, and Sb were extremely significantly positively correlated with the nickelalumite and bearsite contents. Additionally, Ni was extremely significantly positively correlated with indium minerals, whereas As was extremely significantly positively correlated with coesite. Mo was extremely significantly positively correlated with nitratine and wyartite. Cd was extremely significantly positively correlated with calcite, mica, and lipcombite and extremely significantly negatively correlated with feldspar. Pb displayed extremely significant positive correlations with gaultite and enstatite.

At the *p* < 0.05 level, Cu was significantly positively correlated with calcite and significantly negatively correlated with feldspar minerals. Sb was significantly positively correlated with steropesite. Mo exhibited significant positive correlations with carlinite and pyroxene. Cd was significantly positively correlated with renierite, magnesium calcite, and taramellite. Pb demonstrated significant negative correlations with quartz and calcite and a significant positive correlation with feldspar.

## 4. Discussion

### 4.1. Spatial Distribution of Heavy Metal Pollution and Its Relationship with Ore Deposits

In terms of the regional mineral distribution pattern in central–western Ali, mining activities and ore deposits are concentrated mainly in Gejie and Gaize counties. The results of the pollution assessments indicate that Gar, Gaize, and Geji are the key areas affected by heavy metal pollution and ecological risks. The elevated heavy metal concentrations in these areas are closely aligned with the distribution of mineral deposits (e.g., chromite and iron ore), suggesting a dominant influence from the natural weathering of mineralized bedrock rather than from intensive mining activities, as most deposits remain unexploited. Additionally, moderate to heavy pollution occurs near magnetite and chromite deposits in Ritu County, partly originating from natural weathering of mineralized zones. Overall, the spatial distribution of mineral deposits fundamentally shapes regional heavy metal pollution patterns. Natural weathering constitutes the primary pollution source, whereas mining acts as a secondary yet significant anthropogenic factor that intensifies contamination.

On the basis of the correlation analysis of heavy metal elements in the surface soils from the central–western Ali region (Figure 6), it can be concluded that the soil pH serves as a key inhibitory factor for the release of Mo and Pb. Under alkaline conditions, these elements are more likely to precipitate and less likely to be mobilized. TK and TN positively influence the activities of Cu, Cd, and Co through biogeochemical processes [39]. A high TOC content promotes the migration of Cd while inhibiting the release of Pb [40,41]. TP may affect Cu behavior via competitive adsorption or precipitation mechanisms [42]. The significant positive correlations (*p* < 0.01) among Cr, Co, Ni, and Cu suggest a common source for these elements. Cu was also positively correlated with Cd, and Cd was positively correlated only with Mo, indicating that Mo, Cu, and Cd likely share a common source. However, Cu may also stem from an additional pollution source that is distinct from those of Cr, Co, Ni, and the Mo–Cd group. Since As was correlated only with Sb and Sb was positively correlated with Cr, Co, and Ni, there may be two distinct factors contributing to Sb pollution.

The PCA of the heavy metal elements in the surface soils from the central–western Ali region revealed that the pollution sources can be categorized into three main components. The first and most significant principal component is dominated by Cr, Ni, Co, and Cu. The abundant ultramafic rock–derived soils in the region result in natural accumulations of these elements [43]. This is because Cr, Co, Ni, and Cu are all iron–loving elements within the fourth–period transition metal group and behave as compatible elements that preferentially accumulate in the crystalline phase, leading to their similar geochemical behavior in surface soils [44]. Therefore, the first principal component primarily reflects the natural geological background, specifically the mineralogy and regional ore deposits, which are the fundamental drivers of elemental migration and enrichment in local soils. As indicated in Table 1, Cd had a high CV value (50–100%) and a mean concentration that was notably above the regional soil background value. Although Cd is sometimes cited as a marker for agricultural activities in other regions [45], no such evidence was found in this study. Elevated Cd levels are more likely attributable to a combination of natural processes (e.g., arid climate and alkaline soil conditions) and nonagricultural anthropogenic influences such as transportation emissions and regional deposition. The arid climate and alkaline soil conditions in the Ali region can significantly affect the mobility and enrichment of elements such as Cd, potentially leading to its accumulation independent of anthropogenic activity. Similarly, Mo is commonly used in automobile oil pumps [46], and Pb pollution has been linked to historical vehicle exhaust from leaded gasoline and long–range atmospheric transport [44,47]. Thus, the second principal component appears to represent a mixed signal, reflecting the combined impact of industrial and vehicular emissions, long–distance atmospheric deposition, and natural processes such as arid climate and alkaline soil conditions that influence Cd mobility. The third principal component is associated with As and Sb. Studies focused on the broader Qinghai region have revealed numerous areas of natural surface As enrichment [48,49], which may form a high geological background in this area. Anthropogenic activities, such as industrial uses of As (e.g., in alloys or semiconductors) [50] and Sb release from fossil fuel combustion, nonferrous metal smelting, and traffic–related emissions [51], could serve as additional, superimposed sources. Given the high CV value of Sb, it is likely influenced by a combination of natural geological variability and anthropogenic pressures such as mining activities and fossil fuel use [38]. Therefore, the third principal component likely represents the superimposed effects of industrial processing, fossil fuel use, and human mining activities on the inherent geological background of soil heavy metals.

### 4.2. Restrictive Effects of Mineral Compositions on Heavy Metal Enrichment and Migration

A correlation analysis between the heavy metal and mineral compositions in the surface soils from the central–western Ali region revealed that the heavy metal contents are closely related to the mineralogy. The general formula of the spinel structure is AB_2×4_, where A and B denote divalent or trivalent metal cations [52], and X is an O^2−^ or S^2−^ ion. Co^3+^, Cr^3+^, and Cu^2+^ can be substituted for B^3+^ in the lattice [53], whereas under oxidizing or reducing conditions, Sb^3+^/Sb^5+^ often becomes adsorbed onto mineral surfaces [54]. Therefore, in nickelalumite, Co^3+^, Cr^3+^, and Cu^2+^ may be substituted for Al^3+^ in the lattice, and Sb^3+^ may be adsorbed under reducing conditions. This finding conforms with the PCA results, indicating that Cr, Co, Ni, and Cu preferentially accumulate in the crystalline phases. Iron arsenic sulfide (FeAsS) contains Cu, Co, Ni, and Sb [55]. Since As and Sb belong to the same group in the periodic table, they are often coenriched in sulfide ore deposits or hydrothermal systems. Early–crystallizing sulfides in pyrrhotite–pentlandite ore deposits frequently include antimony minerals such as stibnite (Pd_3_Sb) alongside arsenoplatinite (PtAsS_2_) [56]. Both Ni^2+^ and In^3+^ are chalcophile elements commonly found in cassiterite–sulfide or massive volcanic sulfide deposits; In is present primarily in sphalerite, involving the substitution of Zn^2+^ with In^3+^. Because sphalerite often coexists with bearsite [57], both Ni^2+^ and In^3+^ are chalcophile elements commonly found in cassiterite–sulfide or massive volcanic sulfide deposits [58,59]. The lack of correlation between As and bearsite may result from the wide dispersal and low concentration of As in bearsite or substitution by other elements. Nitratine forms under arid, strongly oxidizing conditions, such as saltwater lakes and desert surfaces, whereas Mo oxides exhibit high solubility, leading to Mo coprecipitation during alkaline evaporation processes [60]. Wyartite (CaU^5+^(MoO_4_)_2_(OH)·3H_2_O) contains Mo, explaining the strong positive correlation between Mo and wyartite. With respect to Cd and calcite, Cd^2+^ (ionic radius ≈ 0.97 Å) can be substituted for Ca^2+^ (≈1.00 Å) within the calcite (CaCO_3_) lattice, forming a solid solution (Cd_x_Ca_1−x_CO_3_) [61]. In feldspar–dominated systems, Cd is adsorbed onto or migrates with other minerals, but it is strongly adsorbed onto mica. Lipscombite, which contains PO_4_^3−^, can react with Cd to form insoluble compounds, thus immobilizing Cd within its structure [62]. Gaultite is a microporous silicate mineral, and Pb^2+^ commonly forms inner–sphere complexes with aluminosilicate minerals such as mica and feldspar and generally exhibits greater adsorption than Cd^2+^ does [62]. A similar adsorption mechanism likely explains the strong positive correlation between Pb and gaultite. Enstatite (MgSiO_3_) can exchange Mg^2+^ ions with Pb^2+^ ions on its surface, contributing to the positive correlation between Pb and enstatite.

Copper minerals may be irregularly or semi–irregularly encapsulated within gangue mineral grains such as quartz, pyroxene, garnet, calcite, and mica [63]. Cu^2+^ is only weakly adsorbed onto feldspar surfaces through outer–sphere complexation, making it easily mobilized by fluids and resulting in relatively low Cu concentrations in feldspar–rich areas [64]. The chemical formula of steropesite is (Mg, Fe)_2_[SiO_4_]. Its antimony content increases with increasing magnesium content, likely because the ionic radii of Sb^3+^ (0.076 nm) and Sb^5+^ (0.062 nm) are similar to those of Fe^2+^ (0.074 nm) and Fe^3+^ (0.064 nm), respectively, facilitating ionic substitution [65]. Carlinite is a copper sulfide mineral (CuS_2_). Previous studies have shown that sulfides such as pyrite (FeS_2_) can strongly adsorb Mo [66], suggesting that carlinite may share similar adsorption mechanisms for Mo. Pyroxene forms under conditions of 1100–1400 °C and 11–15 kbar, corresponding to depths of 33–45 km in the lower crust or magma chamber, favoring the partitioning of Mo from the melt into fluid, which then precipitates at shallower levels as molybdenite (MoS_2_) [67]. Renierite is a complex sulfosalt mineral containing Cu, Zn, Ge, As, and Fe. Cd^2+^ (ionic radius ≈ 0.95 Å) can be substituted for Zn^2+^ (0.74 Å) or Fe^2+^ (0.78 Å) in the mineral lattice, leading to Cd enrichment in renierite [68]. Magnesium calcite is a (Ca, Mg)CO_3_ solid solution system; the incorporation of Mg^2+^ (0.72 Å) causes lattice contraction and enhances Cd^2+^ (0.97 Å) substitution for Ca^2+^ (1.00 Å), resulting in Cd enrichment within magnesium calcite [69]. Taramellite, which is a layered silicate mineral, forms inner–sphere complexes with Cd, similar to the mechanisms observed for Pb and gaultite [62]. Quartz surfaces are dominated by ≡Si–O^−^ groups but lack active sites such as ≡Al–O^−^ found in feldspar; thus, quartz has a very low adsorption capacity for Pb^2+^ because of electrostatic repulsion, whereas feldspar exhibits much greater Pb^2+^ adsorption [62]. The significant negative correlation observed between calcite and Pb deviates from existing research and may be explained by Pb occurring as an independent mineral phase (e.g., PbCO_3_) at alkaline sampling sites rather than being adsorbed onto calcite surfaces.

In addition to the crystalline phases that were identified by XRD, the scavenging of heavy metals in soils is often dominated by amorphous or poorly crystalline materials, such as hydrous ferric and aluminum oxides and allophane, which escape detection by standard diffraction techniques [70]. These materials possess high specific surface areas and reactive surface sites (e.g., hydroxyl groups) that facilitate the immobilization of metals through adsorption and surface complexation [71]. Furthermore, organic matter, particularly humic substances, plays a crucial role as an effective scavenger by forming stable complexes with metal ions because of its abundant functional groups (e.g., carboxylic and phenolic groups) [71]. The significant contributions of these ‘hidden’ phases likely explain the observed low bioavailability and leachability of heavy metals in the present study, despite the absence of identifiable primary heavy metal minerals in the XRD patterns [71].

## 5. Conclusions

In this study, the pollution status, spatial distribution, sources, and ecological risks of nine heavy metals in surface soils from the central–western Ali region of the Tibetan Plateau were systematically characterized. The results demonstrated that heavy metal pollution exhibits significant spatial heterogeneity, with elevated concentrations of Cr, Ni, Co, and Cu being closely associated with the distributions of ultramafic rocks and Fe–Cr deposits, indicating a dominant natural origin from bedrock weathering. In contrast, the anthropogenic influences of As, Cd, Mo, Pb, and Sb were clearly linked to mining, transportation, and industrial activities.

Multivariate analyses revealed three primary sources: natural weathering (Cr–Ni–Co–Cu); a mixed source for Cd–Mo–Pb influenced by vehicular emissions and long–range atmospheric transport; and a combined source for As–Sb reflecting both the high geological background and anthropogenic inputs from mining and fossil fuel use. Mineralogical analyses further revealed that heavy metals are retained in soils through lattice substitution in spinel minerals, adsorption on sulfides and silicates, and ionic exchange in carbonates.

Environmental factors such as soil alkalinity, total organic carbon, and nutrient content significantly influence the mobility and bioavailability of heavy metals. Specifically, alkaline conditions promoted the immobilization of Mo and Pb, whereas organic matter enhanced Cd mobility but stabilized Pb.

These findings provide a scientific basis for targeted pollution control strategies in alpine ecological barrier regions. Priority should be given to monitoring Cd dynamics and controlling As and Sb emissions in areas that are affected by mining and transport activities and enhancing monitoring in ecologically sensitive zones such as saline lake peripheries and areas with high mineral deposit density.

## Figures and Tables

**Table 1:** Descriptive statistics of heavy metals in surface soil in the central and western Ali region.

	Minimum Value (mg·kg^−1^)	Maximum Value (mg·kg^−1^)	Average Value (mg·kg^−1^)	Coefficient of Variation (%)	Skewness	Kurtosis	UCC [36] (mg·kg^−1^)	Risk–Based Screening Level [37] (mg·kg^−1^) (pH > 7.5)	Soil Background Value [30] (mg·kg^−1^)
Cr	12.5	370.1	82.4	80.0	2.4	6.7	35	250	77.4
Co	2.8	20.8	8.6	35.4	1.3	3.9	10	—	11.6
Ni	5.2	135	37.3	76.6	2.1	4.3	20	190	32.1
Cu	4.8	30.3	13.9	44.6	0.7	0.1	25	100	21.9
As	3.2	186	22.3	111	5.7	37.1	1.5	25	18.7
Mo	0.2	1.7	0.7	42.9	1.2	1.3	1.45	—	1.14
Cd	0.04	0.3	0.1	53.1	1.4	2.4	0.098	0.6	0.08
Sb	0.4	5.8	1.5	71.9	2.5	6.8	0.2	—	1.69
Pb	11.5	41.2	21.0	27.0	1.1	1.9	20	250	28.9

## Data Availability

The original contributions presented in this study are included in the article/Supplementary Material. Further inquiries can be directed to the corresponding authors.

## Supplementary Materials

The following supporting information can be downloaded at https://www.mdpi.com/article/10.3390/toxics13110972/s1; Figure S1: Cluster analysis of mineral composition, Figure S2: Proportions of different mineral classifications, Figure S3: Spatial distribution of geoaccumulation index of heavy metals in surface soils in the central and western Ali region ($I_{geo}$), Figure S4: PCA loading plot, Table S1: Evaluation grades for heavy metal pollution, Table S2: Heavy metal risk assessment, Table S3: Nemerow integrated pollution index and mean values, and Table S4: Principal components of heavy metal elements in surface soils of the central and western Ali region.

## References

[1] Hossain M.A., Piyatida P., da Silva J.A.T., Fujita M. (2012). Molecular mechanism of heavy metal toxicity and tolerance in plants: Central role of glutathione in detoxification of reactive oxygen species and methylglyoxal and in heavy metal chelation. J. Bot..

[2] Li Z., Ma Z., van der Kuijp T.J., Yuan Z., Huang L. (2014). A review of soil heavy metal pollution from mines in China: Pollution and health risk assessment. Sci. Total Environ..

[3] Wuana R.A., Okieimen F.E. (2011). Heavy metals in contaminated soils: A review of sources, chemistry, risks and best available strategies for remediation. Int. Sch. Res. Not..

[4] Rajendran S., Priya T.A.K., Khoo K.S., Hoang T.K., Ng H.S., Munawaroh H.S.H., Karaman C., Orooji Y., Show P.L. (2022). A critical review on various remediation approaches for heavy metal contaminants removal from contaminated soils. Chemosphere.

[5] Rehman Z.U., Sardar K.H.A.N., Shah M.T., Brusseau M.L., Khan S.A., Mainhagu J. (2018). Transfer of heavy metals from soils to vegetables and associated human health risks at selected sites in Pakistan. Pedosphere.

[6] Štrbac S., Ranđelović D., Gajica G., Hukić E., Stojadinović S., Veselinović G., Orlić J., Tognetti R., Kašanin-Grubin M. (2022). Spatial distribution and source identification of heavy metals in European mountain beech forests soils. Chemosphere.

[7] Wu Y., Bin H., Zhou J., Luo J., Yu D., Sun S., Li W. (2011). Atmospheric deposition of Cd accumulated in the montane soil, Gongga Mt. China. J. Soils Sediments.

[8] Zeng S., Li X., Yang L., Wang D. (2023). Understanding heavy metal distribution in timberline vegetations: A case from the Gongga Mountain, eastern Tibetan Plateau. Sci. Total Environ..

[9] Smith-Downey N.V., Sunderland E.M., Jacob D.J. (2010). Anthropogenic impacts on global storage and emissions of mercury from terrestrial soils: Insights from a new global model. J. Geophys. Res. Biogeosci..

[10] Bindler R. (2011). Contaminated lead environments of man: Reviewing the lead isotopic evidence in sediments, peat, and soils for the temporal and spatial patterns of atmospheric lead pollution in Sweden. Environ. Geochem. Health.

[11] Bing H., Wu Y., Li J., Xiang Z., Luo X., Zhou J., Sun H., Zhang G. (2019). Biomonitoring trace element contamination impacted by atmospheric deposition in China’s remote mountains. Atmos. Res..

[12] Moser K.A., Baron J.S., Brahney J., Oleksy I.A., Saros J.E., Hundey E.J., Sadro S., Kopáček J., Sommaruga R., Kainz M.J. (2019). Mountain lakes: Eyes on global environmental change. Glob. Planet. Change.

[13] Li Z., He Y., Yang X., Theakstone W.H., Jia W., Pu T., Liu Q., He X., Song B., Zhang N. (2010). Changes of the Hailuogou glacier, Mt. Gongga, China, against the background of climate change during the Holocene. Quat. Int..

[14] Arellano L., Fernández P., Tatosova J., Stuchlik E., Grimalt J.O. (2011). Long-range transported atmospheric pollutants in snowpacks accumulated at different altitudes in the Tatra Mountains (Slovakia). Environ. Sci. Technol..

[15] Chen Y., Weng L., Ma J., Wu X., Li Y. (2019). Review on the last ten years of research on source identification of heavy metal pollution in soils. J. Agro-Environ. Sci..

[16] Liu X., Gao W., Wei T., Dong Z., Ren J., Shao Y., Chen X. (2024). Distribution and source of heavy metals in Tibetan Plateau topsoil: New insight into the influence of long-range transported sources to the surrounding glaciers. Environ. Pollut..

[17] Wang Y., Cheng H. (2023). Soil heavy metal (loid) pollution and health risk assessment of farmlands developed on two different terrains on the Tibetan Plateau, China. Chemosphere.

[18] Zhang S., Yang G., Hou S., Zhang T., Li Z., Du W. (2021). Analysis of heavy metal-related indices in the Eboling permafrost on the Tibetan Plateau. Catena.

[19] You Q., Chen D., Wu F., Pepin N., Cai Z., Ahrens B., Jiang Z., Wu Z., Kang S., AghaKouchak A. (2020). Elevation dependent warming over the Tibetan Plateau: Patterns, mechanisms and perspectives. Earth-Sci. Rev..

[20] Skierszkan E.K., Dockrey J.W., Lindsay M.B. (2024). Metal Mobilization from Thawing Permafrost is an Emergent Risk to Water Resources. ACS EST Water.

[21] Zhao Z., Li T., Zhang Y., Lü D., Wang C., Lü Y., Wu X. (2022). Spatiotemporal patterns and driving factors of ecological vulnerability on the Qinghai-Tibet Plateau based on the google earth engine. Remote Sens..

[22] Muller G. (1969). Index of geoaccumulation in sediments of the Rhine River. Sci. Inf. Database.

[23] Tomlinson D.L., Wilson J.G., Harris C.R., Jeffrey D.W. (1980). Problems in the assessment of heavy-metal levels in estuaries and the formation of a pollution index. Helgoländer Meeresunters.

[24] Bhuyan M.S., Bakar M.A., Rashed-Un-Nabi M., Senapathi V., Chung S.Y., Islam M.S. (2019). Monitoring and assessment of heavy metal contamination in surface water and sediment of the Old Brahmaputra River, Bangladesh. Appl. Water Sci..

[25] Hakanson L. (1980). An ecological risk index for aquatic pollution control. A Sedimentological approach. Water Res..

[26] Chen R., Cai X., Ding G., Ren F., Wang Q., Cheng N., Liu J., Li L., Shi R. (2021). Ecological risk assessment of heavy metals in farmland soils in Beijing by three improved risk assessment methods. Environ. Sci. Pollut. Res. Int. Nov..

[27] Petrosyan V., Pirumyan G., Perikhanyan Y. (2019). Determination of heavy metal background concentration in bottom sediment and risk assessment of sediment pollution by heavy metals in the Hrazdan River (Armenia). Appl. Water Sci..

[28] Wei-Xin L.I., Zhang X.X., Bing W.U., Shi-Lei S.U.N., Yan-Song C.H.E.N., Wen-Yang P.A.N., Da-Yong Z.H.A.O., Cheng S.P. (2008). A comparative analysis of environmental quality assessment methods for heavy metal-contaminated soils. Pedosphere.

[29] Chen X., Li F., Zhang J., Liu S., Ou C., Yan J., Sun T. (2021). Status, fuzzy integrated risk assessment, and hierarchical risk management of soil heavy metals across China: A systematic review. Sci. Total Environ..

[30] Cheng Y.A., Tian J.L. (1993). Background Values of Soil Elements and Their Distribution Characteristics in Tibet.

[31] Han R., Zhou B., Huang Y., Lu X., Li S., Li N. (2020). Bibliometric overview of research trends on heavy metal health risks and impacts in 1989–2018. J. Clean. Prod..

[32] Adewumi A.J., Ogundele O.D. (2024). Hidden hazards in urban soils: A meta-analysis review of global heavy metal contamination (2010–2022), sources and its Ecological and health consequences. Sustain. Environ..

[33] Pekey H., Karakaş D., Bakoglu M. (2004). Source apportionment of trace metals in surface waters of a polluted stream using multivariate statistical analyses. Mar. Pollut. Bull..

[34] Li Q.Y., Wei M.H., Dai H.M., He P.F., Liu K. (2021). Characteristics of soil heavy metal pollution and ecological risk assessment in Jinzhou City. Geol. Resour..

[35] Garcıa-Sánchez A., Alastuey A., Querol X. (1999). Heavy metal adsorption by different minerals: Application to the remediation of polluted soils. Sci. Total Environ..

[36] Taylor S.R., McLennan S.M. (1995). The geochemical evolution of the continental crust. Rev. Geophys..

[37] (2018). Ministry of Ecology and Environment of the People’s Republic of China, State Administration for Market Regulation, Soil Environmental Quality—Risk Control Standard for Soil Contamination of Agricultural Land (Trial).

[38] Wilson S.C., Lockwood P.V., Ashley P.M., Tighe M. (2010). The chemistry and behaviour of antimony in the soil environment with comparisons to arsenic: A critical review. Environ. Pollut..

[39] Guo G., Wu F., Xie F., Zhang R. (2012). Spatial distribution and pollution assessment of heavy metals in urban soils from southwest China. J. Environ. Sci..

[40] Luo H., Du P., Wang P., Chen J., Li Y., Wang H., Teng Y., Li F. (2022). Chemodiversity of dissolved organic matter in cadmium-contaminated paddy soil amended with different materials. Sci. Total Environ..

[41] Ge S., Pan Y., Zheng L., Xie X. (2020). Effects of organic matter components and incubation on the cement-based stabilization/solidification characteristics of lead-contaminated soil. Chemosphere.

[42] Zhou L.X., Wong J.W.C. (2001). Effect of dissolved organic matter from sludge and sludge compost on soil copper sorption. J. Environ. Qual..

[43] Rudnick R.L., Gao S. (2005). Composition of the continental crust. The Crust. Treatise on Geochemistry.

[44] Nriagu J.O. (1989). A global assessment of natural sources of atmospheric trace metals. Nature.

[45] Roberts T.L. (2014). Cadmium and phosphorous fertilizers: The issues and the science. Procedia Eng..

[46] Barbosa J.Z., Poggere G.C., Teixeira W.W.R., Motta A.C.V., Prior S.A., Curi N. (2020). Assessing soil contamination in automobile scrap yards by portable X-ray fluorescence spectrometry and magnetic susceptibility. Environ. Monit. Assess..

[47] Yuan H., Liu E., Shen J., Zhou H., Geng Q., An S. (2014). Characteristics and origins of heavy metals in sediments from Ximen Co Lake during summer monsoon season, a deep lake on the eastern Tibetan Plateau. J. Geochem. Explor..

[48] Li C., Kang S., Zhang Q. (2009). Elemental composition of Tibetan Plateau top soils and its effect on evaluating atmospheric pollution transport. Environ. Pollut..

[49] Sheng J., Wang X., Gong P., Tian L., Yao T. (2012). Heavy metals of the Tibetan top soils: Level, source, spatial distribution, temporal variation and risk assessment. Environ. Sci. Pollut. Res..

[50] Mandal B.K., Suzuki K.T. (2002). Arsenic round the world: A review. Talanta.

[51] Wei T., Dong Z., Kang S., Zong C., Rostami M., Shao Y. (2019). Atmospheric deposition and contamination of trace elements in snowpacks of mountain glaciers in the northeastern Tibetan Plateau. Sci. Total Environ..

[52] Donnay J.D.H. (1965). An Introduction to Crystal Chemistry. J. Am. Chem. Soc..

[53] O’Neill H.S.C., Navrotsky A. (1983). Simple spinels; crystallographic parameters, cation radii, lattice energies, and cation distribution. Am. Mineral..

[54] Wang D., Mathur R., Zheng Y., Qiu K., Wu H. (2021). Redox-controlled antimony isotope fractionation in the epithermal system: New insights from a multiple metal stable isotopic combination study of the Zhaxikang Sb–Pb–Zn–Ag deposit in Southern Tibet. Chem. Geol..

[55] Hazarika P., Mishra B., Pruseth K.L. (2017). Trace-element geochemistry of pyrite and arsenopyrite: Ore genetic implications for late Archean orogenic gold deposits in southern India. Mineral. Mag..

[56] Sluzhenikin S.F., Krivolutskaya N.A., Rad’ko V.A., Malitch K.N., Distler V.V., Fedorenko V.A. Ultramafic-mafic intrusions, volcanic rocks and PGE-Cu-Ni sulfide deposits of the Noril’sk Province, Polar Siberia. Proceedings of the 12th International Platinum.

[57] Murakami H., Ishihara S. (2013). Trace elements of Indium-bearing sphalerite from tin-polymetallic deposits in Bolivia, China and Japan: A femto-second LA-ICPMS study. Ore Geol. Rev..

[58] Chopin C. (1984). Coesite and pure pyrope in high-grade blueschists of the Western Alps: A first record and some consequences. Contrib. Mineral. Petrol..

[59] Zheng Y.F., Gao T.S., Wu Y.B., Gong B., Liu X.M. (2007). Fluid flow during exhumation of deeply subducted continental crust: Zircon U-Pb age and O-isotope studies of a quartz vein within ultrahigh-pressure eclogite. J. Metamorph. Geol..

[60] Risacher F., Alonso H., Salazar C. (2003). The origin of brines and salts in Chilean salars: A hydrochemical review. Earth-Sci. Rev..

[61] Cubillas P., Higgins S.R. (2009). Friction characteristics of Cd-rich carbonate films on calcite surfaces: Implications for compositional differentiation at the nanometer scale. Geochem. Trans..

[62] Farquhar M.L., Vaughan D.J., Hughes C.R., Charnock J.M., England K.E. (1997). Experimental studies of the interaction of aqueous metal cations with mineral substrates: Lead, cadmium, and copper with perthitic feldspar, muscovite, and biotite. Geochim. Cosmochim. Acta.

[63] Stern C.R., Skewes M.A., Arévalo A. (2011). Magmatic evolution of the giant El Teniente Cu–Mo deposit, central Chile. J. Petrol..

[64] Li Y., Audétat A. (2012). Partitioning of V, Mn, Co, Ni, Cu, Zn, As, Mo, Ag, Sn, Sb, W, Au, Pb, and Bi between sulfide phases and hydrous basanite melt at upper mantle conditions. Earth Planet. Sci. Lett..

[65] Grant K.J., Wood B.J. (2010). Experimental study of the incorporation of Li, Sc, Al and other trace elements into olivine. Geochim. Cosmochim. Acta.

[66] Xu N., Christodoulatos C., Braida W. (2006). Adsorption of molybdate and tetrathiomolybdate onto pyrite and goethite: Effect of pH and competitive anions. Chemosphere.

[67] Kamali A.A., Moayyed M., Saumur B.M., Fadaeian M. (2022). Mineralogy and mineral chemistry of dioritic dykes, quartz diorite enclaves and pyroxene of the Sungun Cu-Mo porphyry deposit, East Azerbaijan, Iran. Minerals.

[68] Moëlo Y., Makovicky E., Mozgova N.N., Jambor J.L., Cook N., Pring A., Paar W., Nickel E.H., Graeser S., Karup-Møller S. (2008). Sulfosalt systematics: A review. Report of the sulfosalt sub-committee of the IMA Commission on Ore Mineralogy. Eur. J. Mineral..

[69] Rimstidt J.D., Balog A., Webb J. (1998). Distribution of trace elements between carbonate minerals and aqueous solutions. Geochim. Cosmochim. Acta.

[70] Manceau A., Marcus M.A., Tamura N. (2002). Quantitative speciation of heavy metals in soils and sediments by synchrotron X-ray techniques. Rev. Miner. Geochem..

[71] Bradl H.B. (2004). Adsorption of heavy metal ions on soils and soils constituents. J. Colloid Interface Sci..

