# Supplementary material for: Characterization, Source Analysis, and Ecological Risk Assessment of Heavy Metal Pollution in Surface Soils from the Central–Western Ali Region on the Tibetan Plateau
Source: Toxics. 2025 Nov 12;13(11):972. doi: 10.3390/toxics13110972 (PMC12656361; doi:10.3390/toxics13110972)
Supplement: Supplementary file 1 [file toxics-13-00972-s001.zip › toxics-3954362-Supplmentary materials.pdf]

Supplementary materials

Figure numbers: 4

Table numbers: 4

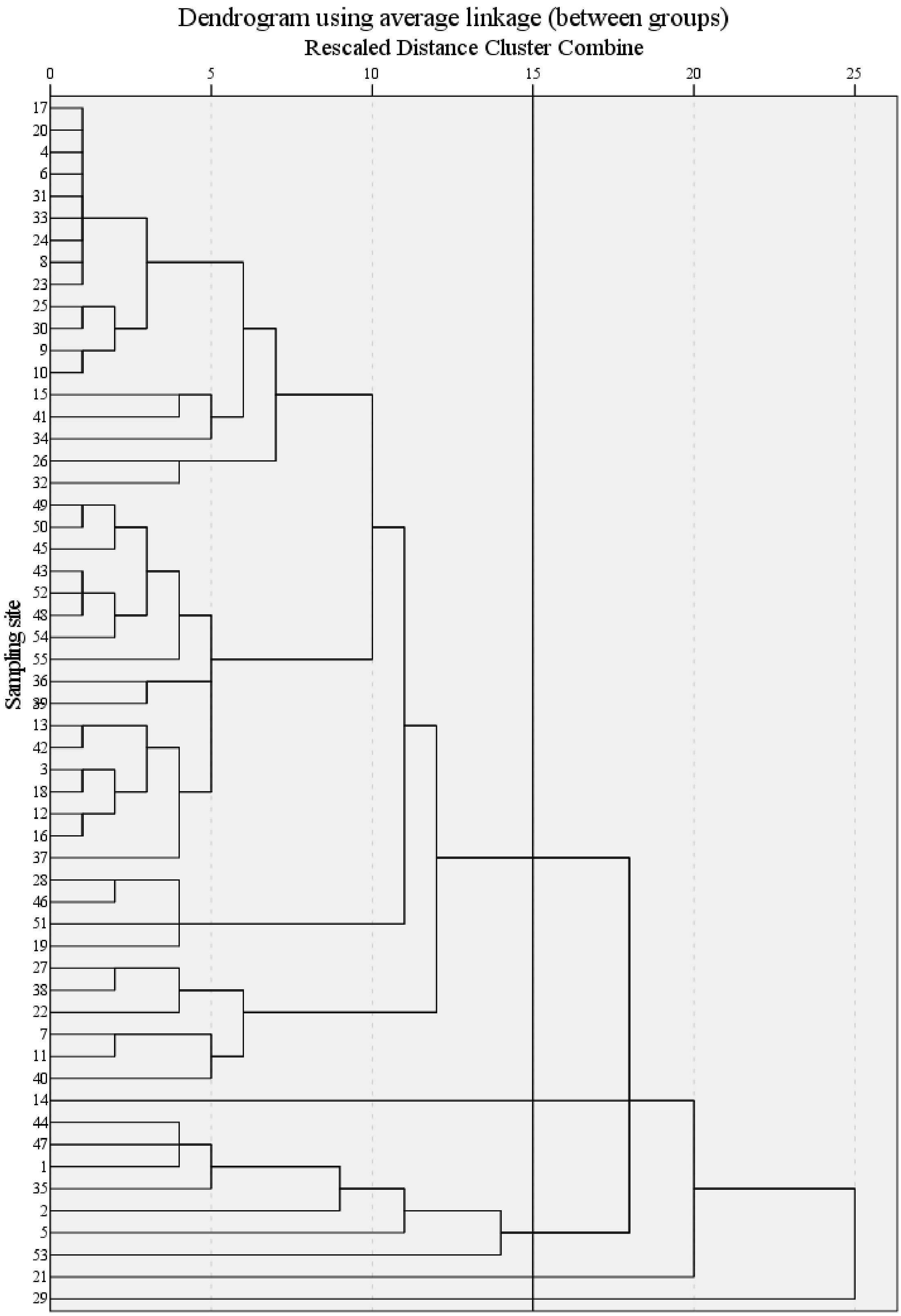

Figure S1 Cluster analysis of mineral composition

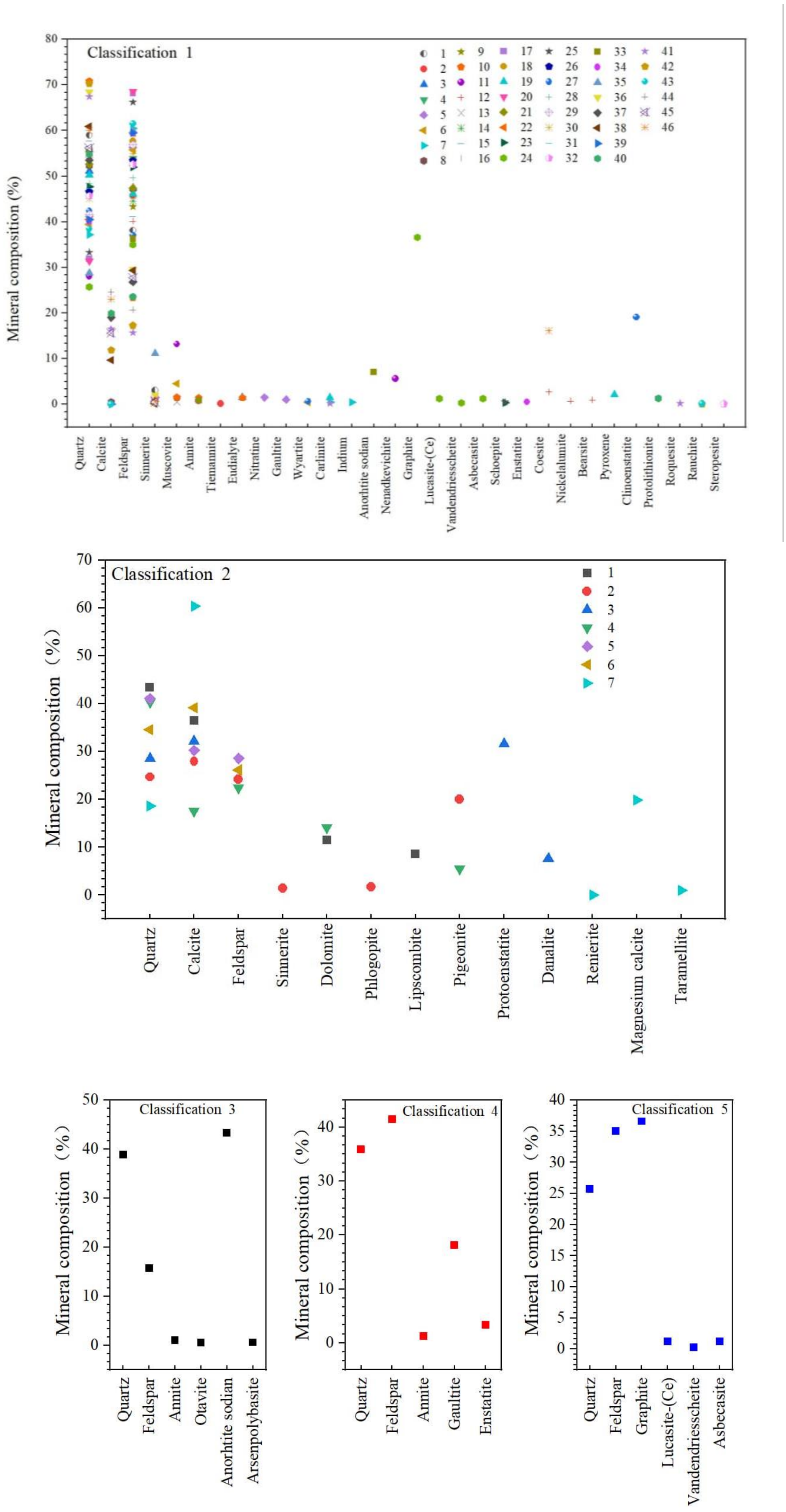

Figure S2 Proportion of different mineral classifications

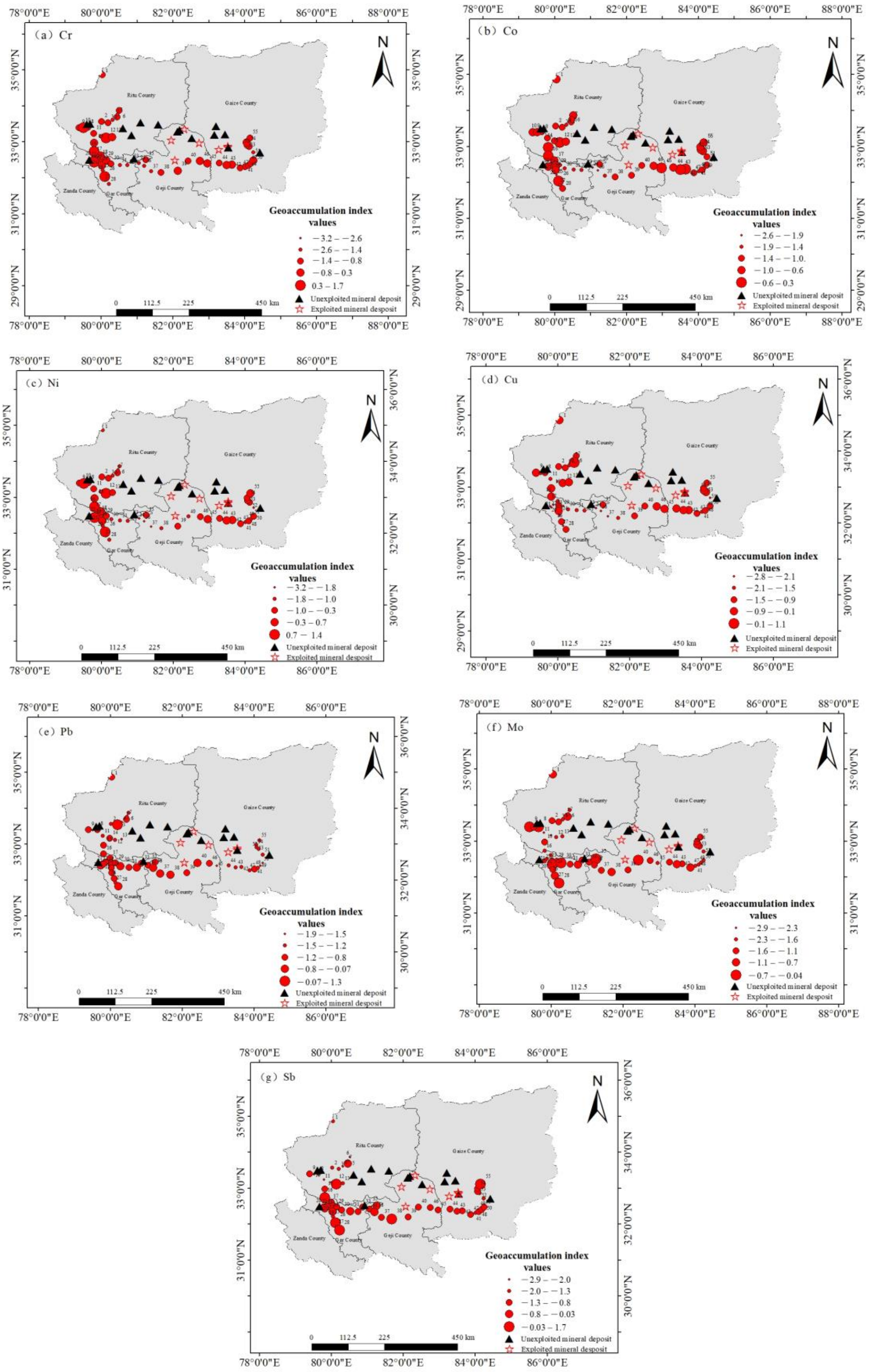

Figure S3 Spatial distribution of geoaccumulation index of heavy metals in surface soils in the central and western Ali region (I_{geo}). (a) Cr, (b) Co, (c) Ni, (d) Cu, (e) Pb, (f) Mo, (g) Sb.

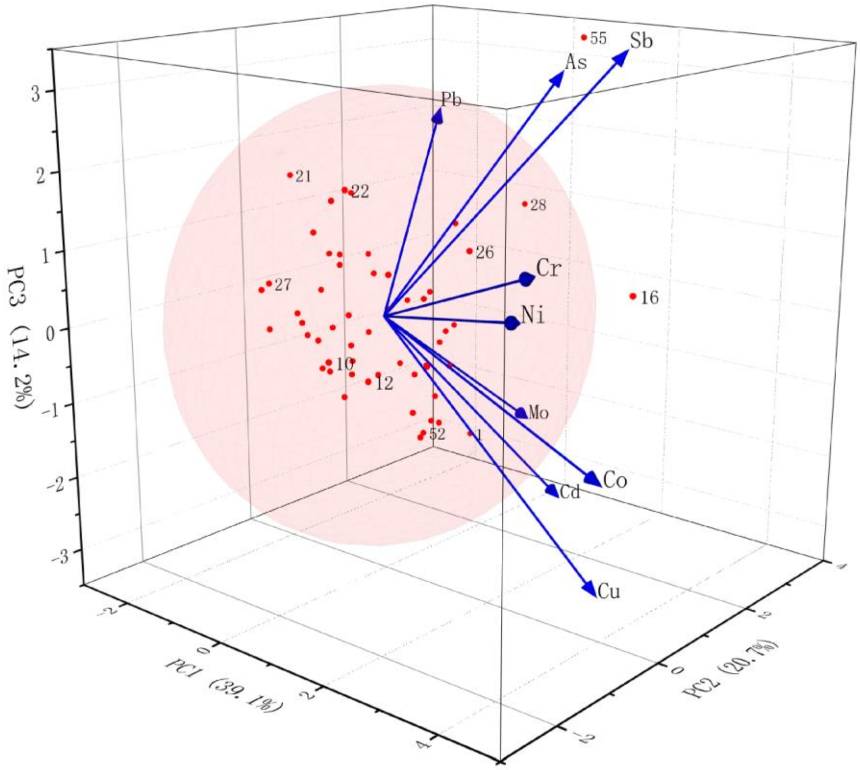

Figure S4 PCA loading plot

Table S1 Evaluation grades for heavy metal pollution

Evaluation index	Formula	Value	Pollution degree
I_{geo}	$I_{geo} = log_2(\frac{C_n}{1.5B_n})$	＜0	Uncontaminated
		0~1	Slightly contaminated
		1~2	Moderately contaminated
		2~3	Moderately to heavily contaminated
		3~4	Heavily contaminated
		4~5	Heavily to extremely contaminated
		≥5	Extremely contaminated
PLI	$CF = C^i_{sample}/C^i_{background}$<br>$PLI = (CF_1 \times CF_2 \times CF_3 \times CF_n)^{1/n}$	PLI≤1	Unpolluted
		1＜PLI≤2	Moderately polluted
		2＜PLI≤3	Heavily polluted
		PLI＞3	Very highly polluted

Note:

The classification criteria for I_{geo} are derived from Muller, 1969

The classification criteria for PLI are derived from Bhuyan et al., 2019

Table S1 Heavy metal risk assessment

E_r^i	RI	Pollution degree (Chen et al., 2021)	Single factor pollution index (Pekey et al., 2004)		Nemerow integrated pollution index (Li et al., 2021)	
E_r^i<40	RI≤110	Low risk	Index	Grade	Index	Grade
40≤E_r^i<80	110＜RI≤220	Moderate risk	P_i≤1	Unpolluted	P≤0.7	Clean
80≤E_r^i<160	220＜RI≤440	Considerable risk	1<P_i≤2	Slightly polluted	0.7＜P≤1.0	Warning line
160≤E_r^i<320	440＜RI≤880	High risk	2<P_i≤3	Moderately polluted	1.0＜P≤2.0	Mild pollution
E_r^i≥320	RI＞880	Very high risk	3<P_i≤5	Strongly polluted	2.0＜P≤3.0	Moderately polluted
—	—	—	P_i>5	Extremely polluted	P>3.0	Heavily polluted

Table S3 Nemerow integrated pollution index and mean values

Item	Cr	Co	Ni	Cu	As	Mo	Cd	Sb	Pb	Mean value
Central and western Ali region	3.5	1.4	3.1	1.1	7.1	1.1	3.1	2.5	1.1	2.0

Table S4 Principal components of heavy metal elements in surface soils of the central and western Ali region

Component	Initial eigenvalues			Sum of squared loadings		
	Total	Percentage of variance	Cumulative %	Total	Percentage of variance	Cumulative %
1	3.5	39.1	39.1	3.5	39.1	39.1
2	1.9	20.7	59.8	1.9	20.7	59.8
3	1.3	14.2	74.0	1.3	14.1	74.0
4	1.0	10.9	84.9			
5	0.7	7.2	92.1			
6	0.4	4.5	96.6			
7	0.2	2.4	99.0			
8	0.05	0.6	99.6			
9	0.04	0.4	100			
